# Dental Neuralgia

**Published:** 1861-02

**Authors:** 


Dental Neuralgia.—M. Balloy prescribes for this:

| | |
|---|---|
| Acetate of morphia, - - - | gr. iss. |
| Acetic acid, - - - - - | gr. ij. |
| Eau de Cologne, - - - - | ℨ ij. |

To be dropped on cotton or wool and placed in the ear on the painful side.

